# Tenuifolide B from *Cinnamomum tenuifolium* Stem Selectively Inhibits Proliferation of Oral Cancer Cells via Apoptosis, ROS Generation, Mitochondrial Depolarization, and DNA Damage

**DOI:** 10.3390/toxins8110319

**Published:** 2016-11-05

**Authors:** Chung-Yi Chen, Ching-Yu Yen, Hui-Ru Wang, Hui-Ping Yang, Jen-Yang Tang, Hurng-Wern Huang, Shih-Hsien Hsu, Hsueh-Wei Chang

**Affiliations:** 1 Department of Nutrition and Health Sciences, School of Medical and Health Sciences, Fooyin University, Kaohsiung 83102, Taiwan; XX377@fy.edu.tw; 2 Department of Oral and Maxillofacial Surgery Chi-Mei Medical Center, Tainan 71004, Taiwan; ycysmc@gmail.com; 3 School of Dentistry, Taipei Medical University, Taipei 11031, Taiwan; 4 Department of Biomedical Science and Environmental Biology, Kaohsiung Medical University, Kaohsiung 80708, Taiwan; whr0319@gmail.com; 5 Graduate Institute of Medicine, College of Medicine, Kaohsiung Medical University, Kaohsiung 80708, Taiwan; kayyang1950@yahoo.com.tw; 6 Department of Radiation Oncology, Faculty of Medicine, College of Medicine, Kaohsiung Medical University, Kaohsiung 80708, Taiwan; reyata@kmu.edu.tw; 7 Department of Radiation Oncology, Kaohsiung Medical University Hospital, Kaohsiung 80708, Taiwan; 8 Department of Radiation Oncology, Kaohsiung Municipal Ta-Tung Hospital, Kaohsiung 80145, Taiwan; 9 Institute of Biomedical Science, National Sun Yat-Sen University, Kaohsiung 80424, Taiwan; sting@mail.nsysu.edu.tw; 10 Institute of Medical Science and Technology, National Sun Yat-sen University, Kaohsiung 80424, Taiwan; 11 Cancer Center, Translational Research Center, Kaohsiung Medical University Hospital, Kaohsiung Medical University, Kaohsiung 80708, Taiwan; 12 Center for Research Resources and Development of Kaohsiung Medical University, Kaohsiung 80708, Taiwan

**Keywords:** selective killing, oral cancer, natural product, apoptosis, ROS, DNA damage

## Abstract

The development of drugs that selectively kill oral cancer cells but are less harmful to normal cells still provide several challenges. In this study, the antioral cancer effects of tenuifolide B (TFB), extracted from the stem of the plant *Cinnamomum tenuifolium* are evaluated in terms of their effects on cancer cell viability, cell cycle analysis, apoptosis, oxidative stress, and DNA damage. Cell viability of oral cancer cells (Ca9-22 and CAL 27) was found to be significantly inhibited by TFB in a dose-responsive manner in terms of ATP assay, yielding IC_50_ = 4.67 and 7.05 μM (24 h), but are less lethal to normal oral cells (HGF-1). Dose-responsive increases in subG1 populations as well as the intensities of flow cytometry-based annexin V/propidium iodide (PI) analysis and pancaspase activity suggested that apoptosis was inducible by TFB in these two types of oral cancer cells. Pretreatment with the apoptosis inhibitor (Z-VAD-FMK) reduced the annexin V intensity of these two TFB-treated oral cancer cells, suggesting that TFB induced apoptosis-mediated cell death to oral cancer cells. Cleaved-poly (ADP-ribose) polymerase (PARP) and cleaved-caspases 3, 8, and 9 were upregulated in these two TFB-treated oral cancer cells over time but less harmful for normal oral HGF-1 cells. Dose-responsive and time-dependent increases in reactive oxygen species (ROS) and decreases in mitochondrial membrane potential (MitoMP) in these two TFB-treated oral cancer cells suggest that TFB may generate oxidative stress as measured by flow cytometry. *N*-acetylcysteine (NAC) pretreatment reduced the TFB-induced ROS generation and further validated that ROS was relevant to TFB-induced cell death. Both flow cytometry and Western blotting demonstrated that the DNA double strand marker γH2AX dose-responsively increased in TFB-treated Ca9-22 cells and time-dependently increased in two TFB-treated oral cancer cells. Taken together, we infer that TFB can selectively inhibit cell proliferation of oral cancer cells through apoptosis, ROS generation, mitochondrial membrane depolarization, and DNA damage.

## 1. Introduction

Oral cancer is the sixth most common type of cancer globally [1] and is especially prevalent in areas that feature a high frequency of betel nut, alcohol, and cigarette consumption [2,3,4]. Oral cancer is likely ignored by patients in early stages and is commonly detected at a later stage. Late detection, combined with poor chemotherapy outcomes, leads to high morbidity and mortality rates of oral cancer [5]. While several drugs have proven effective at killing cancer cells, they are also toxic to normal tissue cells, and the need for selective antioral cancer drugs remains urgent.

A growing number of studies have reported that natural products are potent resources for anticancer drug discovery [6,7,8,9,10,11]. Many bioactive extracts and isolated compounds have been extracted from the bark of *Cinnamomum* of the Formosan Lauraceous family (*C. zeylanicum* and *C. cassia*) [12], leaves (*C. wilsonii* [13], *C. kotoense* [14,15,16,17], *C. subavenium* [18]), stems (*C. subavenium* [19,20]), and heartwood and roots (*C. osmophloeum* [21]). These findings indicate the antiproliferative effect of *Cinnamomum* plants for several types of cancer, such as that of the colon [12,13,17], lung [14,16], liver [15,21], breast [17], prostate [18,20], melanoma [19], and bladder [20]. However, the selective killing effect of *Cinnamomum* plants on oral cancer cells remains undetermined.

To try to discover new compounds from other *Cinnamomum* plants, we extracted material from *C. tenuifolium* Sugimoto form. nervosum (Meissn.) Hara [22], an evergreen form of the Lauraceae plant family grown on Orchid Island of Taiwan. Methanol extracts were used to identify a new benzodioxocinone, benzodioxocinone (2,3-dihydro-6,6-dimethylbenzo-[b][1,5]dioxocin-4(6*H*)-one), from the leaves of *C. tenuifolium* [23]. The benzodioxocinone showed mild levels of cytotoxicity for human oral cancer (OC2), with an IC_50_ value of 107.7 μM after 24 h of treatment.

Alternatively, we previously used the stems of *C. tenuifolium* [22] to identify several novel compounds, including tenuifolide A, isotenuifolide A, tenuifolide B (TFB), secotenuifolide A, and tenuifolin, along with some known compounds. Secotenuifolide A was found to provide the best antiproliferative effect against two human prostate cancer cells (DU145 and LNCaP) with IC_50_ values < 7 μM after 24 h of treatment. For TFB (3-(1-methoxyeicosyl)-5-methylene-5*H*-furan-2-one), its IC_50_ values were 246 and 22.2 μM for DU145 and LNCaP cancer cells after 24 h of treatment [22]. However, the biological effect of TFB against the oral cancer cells was not addressed as yet.

The current study first evaluates the possible selectively antiproliferactive effect and the mechanism of *C. tenuifolium* stem-derived TFB on oral cancer cells by analyzing cell viability, cell cycle progression, apoptosis, reactive oxygen species (ROS) induction, mitochondrial depolarization, and DNA damage.

## 2. Results

### 2.1. Cell Viability and ATP Cellular Content

**Figure 1:**
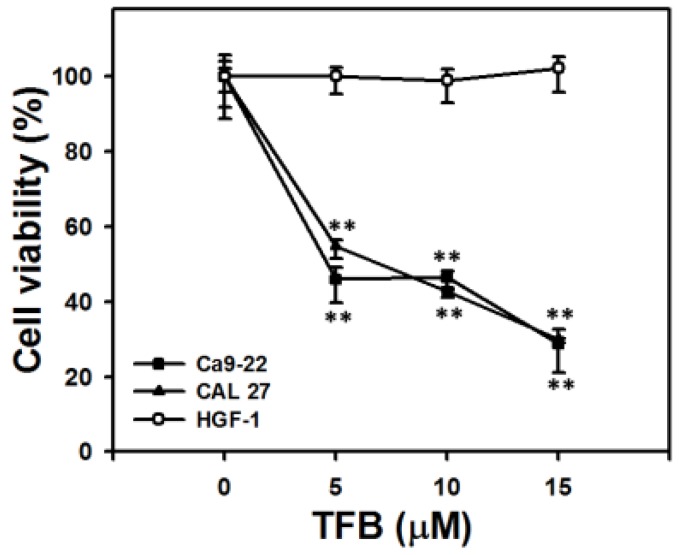
Tenuifolide B (TFB) induced a significant decrease in ATP-based cell viability in oral cancer cells (Ca9-22 and CAL 27) but not in normal oral cells (HGF-1). Cells were treated with 0, 5, 10, and 15 μM TFB for 24 h. Data: mean ± SD (*n* = 4). ** *p* < 0.001 compared to the control.

ATP content has been widely used to measure cell viability [24,25]. Figure 1 shows the ATP assay of cell viability after 24 h of treatment with TFB (0, 5, 10, and 15 μM). The viability of TFB-treated oral cancer cells (Ca9-22 and CAL 27) decreased dose-responsively (*p* < 0.001). In contrast, the normal oral cells (HGF-1) maintained a cell viability of about 100%.

### 2.2. Cell Cycle Progression

**Figure 2:**
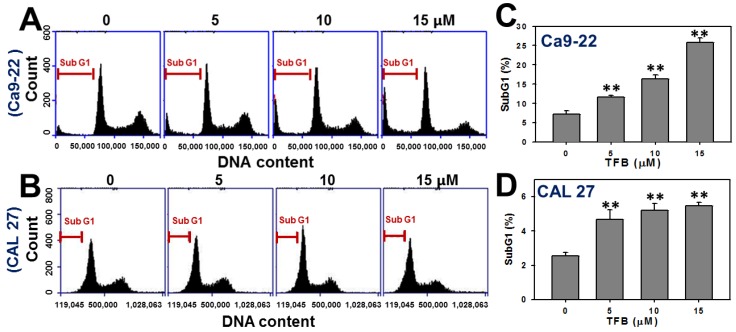
TFB induced an increase in the subG1 population in oral cancer Ca9-22 and CAL 27 cells. (**A**,**B**) Representative dose responses of cell phase profiles in TFB-treated Ca9-22 and CAL 27 cells using flow cytometry. Cells were treated with 0, 5, 10, and 15 μM TFB for 24 h. (**C**,**D**) Quantification analysis results for subG1 population in (**A**,**B**). Data: mean ± SD (*n* = 3). ** *p* < 0.001 compared to the control.

To examine whether the cell cycle was affected by TFB, the cell cycle progression was examined. Figure 2A,B show dose-responsive pattern changes of the cell cycle progression of TFB-treated Ca9-22 and CAL 27 cells, respectively. The subG1 population in TFB-treated Ca9-22 and CAL 27 cells increased in a dose-responsive manner after 24 h of THB treatment (Figure 2C,D) (*p* < 0.001).

### 2.3. Annexin V-Based Apoptosis

**Figure 3:**
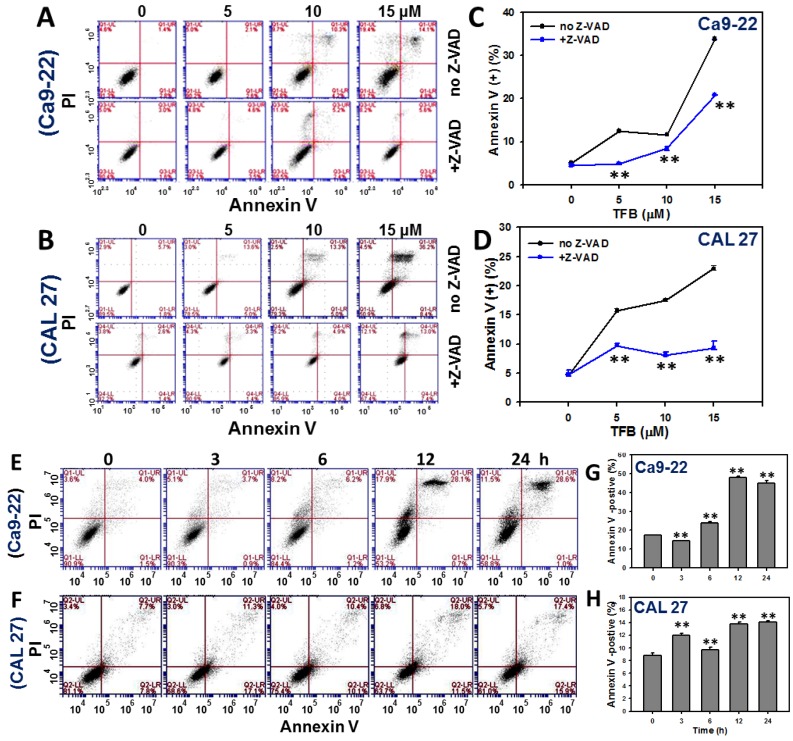
TFB induced annexin V-based apoptosis in oral cancer Ca9-22 and CAL 27 cells. (**A**,**B**) Representative flow cytometry-based dose response of apoptosis profiles of annexin V/PI double staining for TFB-treated Ca9-22 and CAL 27 cells. Cells were treated with 0, 5, 10, and 15 μM TFB for 24 h with or without 0.1 mM Z-VAD-FMK treatment for 2 h. (**C**,**D**) Quantification analysis results for apoptosis positive (%) in (**A**,**B**). Data: mean ± SD (*n* = 3). ** *p* < 0.001, comparing the Z-VAD-FMK to the non-Z-VAD-FMK for each TFB dose. (**E**,**F**) Representative time course of annexin V-based apoptosis profile in TFB-treated Ca9-22 and CAL 27 cells using flow cytometry. Cells were treated with 15 μM TFB for 3, 6, 12, and 24 h. (**G**,**H**) Quantification analysis results for annexin V-based apoptosis in (E,F). The regions of annexin V (+) including annexin V (+)/PI (+) and annexin V (+)/PI (−) were analyzed. Data: mean ± SD (*n* = 3). ** *p* < 0.001 compared to the control.

To validate the role of apoptosis in the increase in the subG1 population in TFB-treated Ca9-22 and CAL 27 cells, the annexin V/propidium iodide (PI) staining method was used. Figure 3A,B respectively show the patterns of dose response changes of annexin V/PI staining profiles of TFB-treated Ca9-22 and CAL 27 cells. By calculating the percentages of annexin V positive (%), the apoptosis level (Figure 3C,D) show a significant increase in a dose-responsive manner in TFB-treated Ca9-22 and CAL 27 cells (*p* < 0.001). When the Ca9-22 and CAL 27 cells were pretreated with apoptosis inhibitor Z-VAD-FMK, apoptosis induced by different doses of TFB was decreased.

Figure 3E,F show the patterns of time course changes of annexin V/PI staining profiles of TFB-treated Ca9-22 and CAL 27 cells. The degree of annexin V/PI staining in TFB-treated Ca9-22 and CAL 27 cells increased in a time-dependent manner (*p* < 0.001) (Figure 3G,H).

### 2.4. Caspases-Based Apoptosis

**Figure 4:**
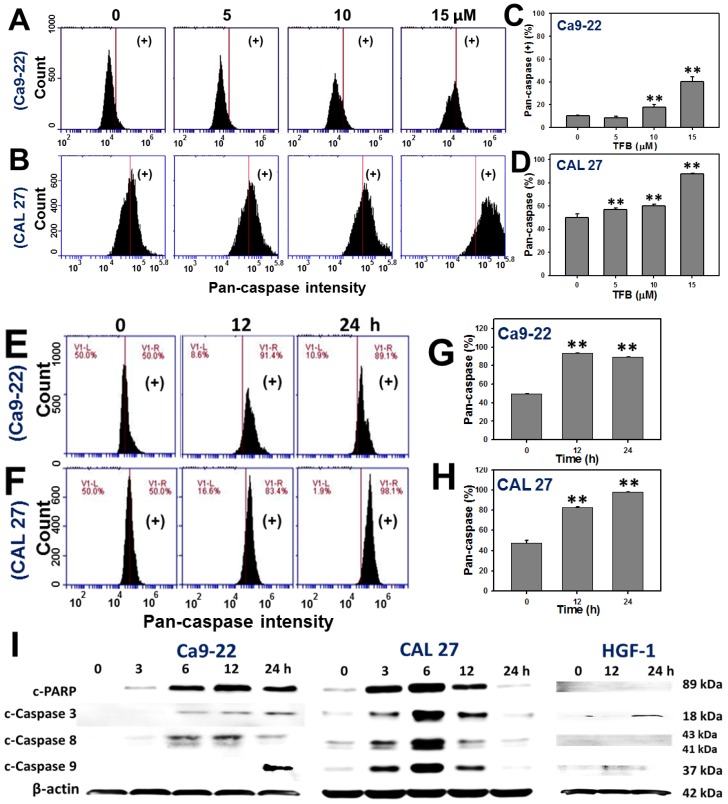
TFB induced caspases-based apoptosis in oral cancer Ca9-22 and CAL 27 cells. (**A**,**B**) Representative flow cytometry-based dose response of apoptosis profiles of pancaspase staining for TFB-treated Ca9-22 and CAL 27 cells. Cells were treated with 0, 5, 10, and 15 μM TFB for 24 h and subsequently stained with TF2-VAD-FMK. (**C**,**D**) Quantification analysis results for pancaspase fluorescent intensity positive (%) in (**A**,**B**). (**E**,**F**) Representative time course of pancaspase-based apoptosis profile in TFB-treated Ca9-22 and CAL 27 cells using flow cytometry. Cells were treated with 15 μM TFB for 12 and 24 h. (**G**,**H**) Quantification analysis results for pancaspase-based apoptosis in (E,F). The regions of pancaspase (+) were analyzed. Data: mean ± SD (*n* = 3). ** *p* < 0.001 compared to the control. (**I**) The apoptosis-related protein expressions in TFB-treated Ca9-22, CAL 27, and HGF-1 cells. Ca9-22 and CAL 27 cells were treated with 15 μM TFB for 3, 6, 12, and 24 h. HGF-1 cells were treated with 15 μM TFB for 12 and 24 h. Apoptosis signaling proteins, such as cleavage forms of PARP, procaspase 3, procaspase 8, and procaspase 9 were detected using Western blotting. The β-actin was used as an internal control.

To validate the role of apoptosis in the increase in annexin V intensity in TFB-treated Ca9-22 and CAL 27 cells, the pancaspase activity assay was used. Figure 4A,B respectively show the dose-responsive pattern changes of pancaspase intensity profiles of TFB (0, 5, 10, and 15 μM)-treated Ca9-22 and CAL 27 cells. For the pancaspase positive (%), Figure 4C,D respectively show a significant increase in the apoptosis expression in TFB-treated Ca9-22 and CAL 27 cells at higher doses (*p* < 0.001).

Figure 4E,F show the patterns of time course changes of pancaspase intensity profiles of TFB-treated Ca9-22 and CAL 27 cells. The pancaspase intensities in TFB-treated Ca9-22 and CAL 27 cells increased in a time-dependent manner (Figure 4G,H) (*p* < 0.001). Moreover, Figure 4I shows the protein expressions of apoptosis signaling proteins, such as cleaved-poly (ADP-ribose) polymerase (PARP) and cleaved-caspases 3 and 8 gradually increased over 3–24 h and cleaved-caspase 9 was detected at 24 h in TFB-treated Ca9-22 cells. In TFB-treated CAL 27 cells, the protein expressions of cleaved-PARP and cleaved-caspases 3, 8, and 9 gradually increased from 3 to 6 h, moderately increased at 12 h, and declined at 24 h. In contrast, these apoptosis signaling proteins in TFB-treated HGF-1 were weak.

### 2.5. ROS

**Figure 5:**
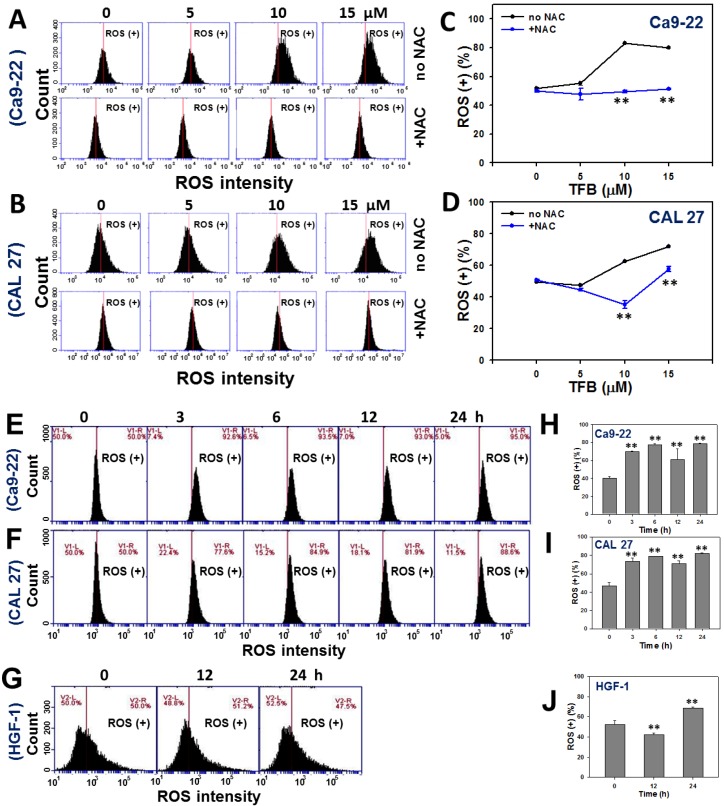
TFB induced ROS generation in oral cancer Ca9-22 and CAL 27 cells. (**A**,**B**) Representative dose responses of ROS profiles for TFB-treated cells using flow cytometry. Cells were treated with 0, 5, 10, and 15 μM of TFB for 24 h with or without 2 mM NAC pretreatment for 1 h. (**C**,**D**) Quantification analysis of ROS intensity for DCFD-A positivity (%). (**E**–**G**) Representative time course of ROS profile in TFB-treated Ca9-22, CAL 27, and HGF-1 cells using flow cytometry. Cells were treated with 15 μM TFB for indicated times. (**H**–**J**) Quantification analysis results for ROS intensity for ROS positivity (%) in (**E**–**G**). Data: mean ± SD (*n* = 3). ** *p* < 0.001 compared to the control.

To determine why TFB may inhibit cancer cell proliferation and induce apoptosis, the cellular ROS level was examined. Figure 5A,B respectively show the dose-responsive pattern changes of ROS change profiles of TFB (0, 5, 10, and 15 μM)-treated Ca9-22 and CAL 27 cells. By calculating the percentages of DCFH-DA fluorescence-positive intensity and adjusting with control, the relative ROS level was increased in a dose-responsive manner in TFB-treated Ca9-22 and CAL 27 cells (*p* < 0.001) (Figure 5C,D). When cells were pretreated by *N*-acetylcysteine (NAC) in Ca9-22 and CAL 27 cells, ROS generation induced by different doses of TFB was decreased.

Figure 5E–G show the time course pattern changes of ROS intensity profiles of TFB-treated Ca9-22, CAL 27, and HGF-1 cells, respectively. The ROS intensities in TFB-treated Ca9-22 and CAL 27 cells dramatically increased in a time-dependent manner (Figure 5H,I) (*p* < 0.001). In contrast, ROS intensities in HGF-1 cells increased only slightly after 24 h of THB treatment (Figure 5J).

### 2.6. Mitochondrial Membrane Potentials (MitoMP)

**Figure 6:**
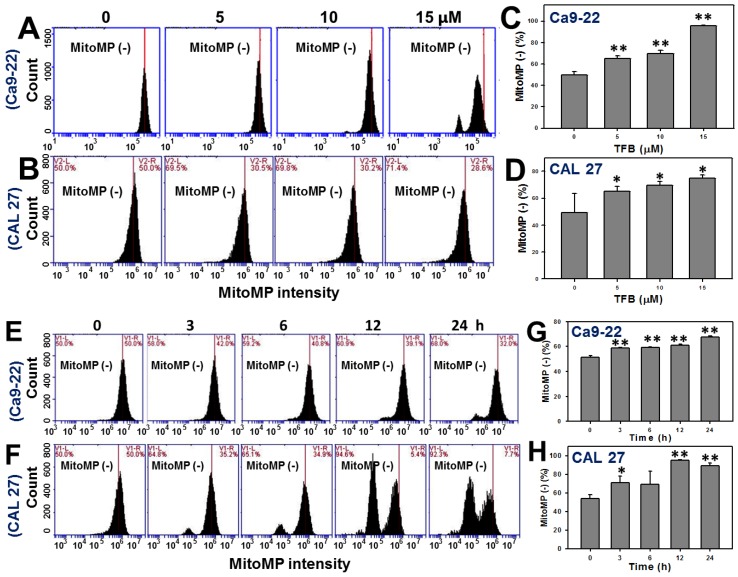
TFB decreased mitochondrial membrane potential (MitoMP) in oral cancer Ca9-22 and CAL 27 cells. (**A**,**B**) Representative dose response of MitoMP profiles for TFB-treated Ca9-22 and CAL 27 cells using flow cytometry. Cells were treated with 0, 5, 10, and 15 μM of TFB for 24 h. (**C**,**D**) Quantification analysis of MitoMP-negative (%) intensity in (A,B). (**E**,**F**) Representative time course of MitoMP profile in TFB-treated Ca9-22 and CAL 27 cells using flow cytometry. Cells were treated with 15 μM TFB for 3, 6, 12, and 24 h. (**G**,**H**) MitoMP-negative (%) intensity in (E,F). The regions of MitoMP-negative (%) were analyzed. Data: mean ± SD (*n* = 3). * *p* < 0.05; ** *p* < 0.001 compared to the control.

DiOC_2_(3)-based MitoMP detection assay was performed to evaluate the impact of TFB (0, 5, 10, and 15 μM)-induced ROS generation. Figure 6A,B show the MitoMP profiles for TFB-treated oral cancer Ca9-22 and CAL 27 cells, respectively, after a 24 h of treatment. By calculating the percentages of DiOC_2_(3)-negative in Figure 6A,B and comparing with the control, it was found that the MitoMP-negative (%) was gradually increased in TFB-treated Ca9-22 and CAL 27 cells in a dose-responsive manner (*p* < 0.001) (Figure 6C,D). Therefore, the MitoMP level of Ca9-22 and CAL 27 cells was significantly decreased after TFB treatment.

Figure 6E,F show the time course pattern changes of MitoMP intensity profiles of TFB-treated Ca9-22 and CAL 27 cells. The MitoMP-negative intensities in TFB-treated Ca9-22 and CAL 27 cells increased in a time-dependent manner (Figure 6G,H) (*p* < 0.001), suggesting that MitoMP levels of Ca9-22 and CAL 27 cells decreased after TFB treatment.

### 2.7. γH2AX Expression

**Figure 7:**
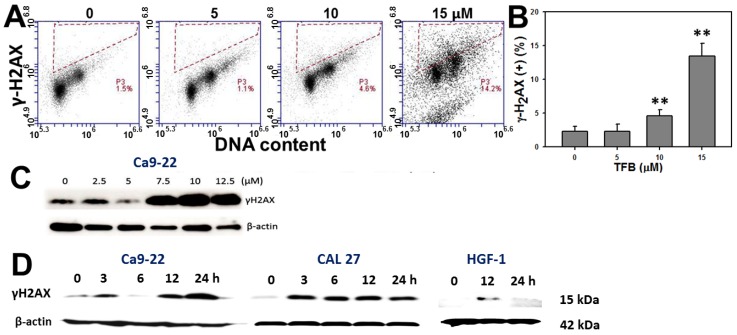
Treatment with TFB induced γH2AX expressions in oral cancer Ca9-22 and CAL 27 cells. (**A**) γH2AX expression of TFB-treated Ca9-22 cells was detected by flow cytometry. Cells were treated with 0, 5, 10, and 15 μM TFB for 24 h. (**B**) Quantification analysis of γH2AX positive (%) intensity of flow cytometry in (**A**). Data: mean ± SD (*n* = 3). ** *p* < 0.001 compared to the control. (**C**) Dose response of γH2AX expression of TFB-treated Ca9-22 cells was detected by Western blotting. The β-actin was used as an internal control. (**D**) Time course of γH2AX expression of TFB-treated Ca9-22, CAL 27, and HGF-1 cells was detected by Western blotting. Oral cancer cells were treated with 15 μM TFB for 0, 3, 6, 12, and 24 h. HGF-1 cells were treated with 15 μM TFB for 12 and 24 h.

To examine the role of DNA damage in TFB-induced antiproliferation of Ca9-22 oral cancer cells, the expression of DNA double strand break marker γH2AX was analyzed via both flow cytometry and Western blotting. Figure 7A shows that the flow cytometry-based γH2AX/PI staining profiles of TFB-treated Ca9-22 cells after 24 h of treatment. Figure 7B shows the γH2AX-positive intensity of TFB (0, 5, 10, and 15 μM)-treated Ca9-22 cells increased in a dose-responsive manner (*p* < 0.001). Moreover, Figure 7C shows that the γH2AX expression by Western blotting of TFB-treated Ca9-22 cells after 24 h of treatment with indicated doses were dramatically increased at higher doses.

For the time course experiments, Figure 7D shows that γH2AX expression of TFB-treated Ca9-22, CAL 27, and HGF-1 cells were increased in a time-dependent manner. The TFB-induced γH2AX levels were dramatically induced in both oral cancer cells (Ca9-22 and CAL 27). In contrast, TFB-induced γH2AX levels increased only slightly at 12 h in oral normal HGF-1 cells.

## 3. Discussion

TFB was previously found to be anti-atherosclerogenic in humans [26], but their possible anticancer effect regarding oral cancer remained unclear. In general, *Cinnamomum* plants generally have antiproliferative effects. For example, the cytotoxicity (IC_50_) is known for several *Cinnamomum* plants. Cinnamaldehyde from *C. zeylanicum* and *C. cassia* barks is effective against colon cancer (HT29) = 19.7 μM at 72 h [12], (3*R*,9*S*)-megastigman-5-ene-3,9-diol 3-*O*-β-d-glucopyranoside from *C. wilsonii* leaves against colon cancer SW-480 cells = 12 μM at 48 h [13], isoobtusilactone A from *C. kotoense* leaves against human hepatoma Hep G2 cells = 37.5 μM at 18 h [15], cinnakotolactone from *C. kotoense* leaves against human colorectal cancer HT29 and breast MCF-7 cells = 25.8 and 24.4 μM at 72 h [17], subamone from *C. subavenium* leaves against prostate cancer LNCaP cells = 7.01 μM at 24 h [18], subamolide B from *C. subavenium* stems against melanoma A375 cells = 17.59 μΜ at 48 h [19], and subamolide A from *C. subavenium* stems against human prostate cancer PC3 cells = 10.1 μM at 72 h [20]. It has to be noted that some of the IC_50_ values were determined after 72 h of treatment.

Based on ATP content assays, the current study found that the IC_50_ values of TFB were 4.67 and 7.05 μM after 24 h of treatment in oral cancer cells (Ca9-22 and CAL 27), respectively. In general, the sensitivity of TFB to oral cancer cells (Ca9-22 and CAL 27) was higher than that of other *Cinnamomum* plants to other types of cancer cells. Moreover, TFB was less cytotoxic to human prostate cancer DU145 and LNCaP cells [22]. These results suggest that TFB may have a cancer cell type-specific antiproliferation effect.

Following 24 h of treatment, the cytotoxicity (IC_50_) of taxol in human prostate cancer DU145 and LNCaP cells is 4.84 and 6.32 μM, respectively [22]. The IC_50_ of cisplatin in oral cancer Ca9-22 cells is 10.2 μM (data not shown). Therefore, our developed TFB (IC_50_ = 4.67 and 7.05 μM) has similar sensitivity to these clinical drugs in oral cancer cells (Ca9-22 and CAL 27). Moreover, we found that TFB is less harmful to normal oral HGF-1 cells (Figure 1), suggesting that TFB selectively kills oral cancer cells and may prevent side effects of oral cancer therapy. Similarly, *Cinnamomum* stem bark extract has been reported to selectively kill other types of cancer cells. In *Cinnamomum burmannii* Blume stem bark extract after 24 h of treatment, the IC_50_ values of nasopharyngeal carcinoma cells (HK1 and C666-1) were 108.32 and 224.32 μg/mL, respectively, whereas the IC_50_ of immortalized human skin keratinocyte HaCaT cells was 320.29 μg/mL [27]. However, our study only tested one normal oral cell line, and further study is needed to confirm these findings, including more normal oral cell lines to further show the lack of possible side-effects of TFB.

Secotenuifolide A, also isolated from the same material of this study (*C. tenuifolium* stems), has been reported to inhibit cell proliferation, increase the subG1 population, induce apoptosis and ROS generation, and decrease mitochondrial membrane potential in human prostate cancer cells, DU145 [22]. Secotenuifolide A also exhibited a release of cytochrome c from mitochondria and the activation of caspase-9/caspase-3 [22]. Similarly, the TFB from *C. tenuifolium* stems showed the same effect of oxidative stress (ROS induction and MitoMP depletion) on oral cancer Ca9-22 and CAL 27 cells (Figure 5 and Figure 6). In contrast, the TFB-induced ROS generation in HGF-1 cells was only slightly induced. These results suggest that TFB exhibited selective ROS induction in oral cancer cells (Ca9-22 and CAL 27), but less induction in HGF-1 cells. NAC pretreatment experiments (Figure 5A,B) validated that ROS was relevant to TFB-induced cell death because the ROS generation in the two types of oral cancer cells was reduced by NAC pretreatment.

Annexin V and pancaspase results (Figure 3 and Figure 4) support that TFB is apoptosis-inducible in oral cancer cells. Moreover, the TFB-induced apoptosis was reduced by Z-VAD-FMK pretreatment (Figure 3A,B), suggesting that apoptosis was involved in selective killing by TFB. However, the role of apoptosis signaling in TFB-induced apoptosis was not addressed specifically. Caspases 8 and 9 involved in intrinsic and extrinsic apoptotic pathways, respectively. Both converge in activating the executioner caspases 3 and 7 [28]. PARP is also involved in apoptosis [29,30]. To address the role of apoptosis signaling, we found that both TFB-treated oral cancer cells (Ca9–22 and CAL 27) induced activation of PARP and caspases 3, 8, and 9 by cleavage. However, these oral cancer cells displayed a differential expression of these TFB-induced apoptosis proteins. For example, cleaved-caspase 8 was mainly or early induced during 3–12 h in TFB-treated Ca9-22 cells, but cleaved-caspase 9 induction showed a later response at 24 h (Figure 4I). For CAL 27 cells, cleaved-PARP and cleaved-caspases 3, 8 and 9 were upregulated early after 3 h. These caspases peaked at 6 h, gradually declining by 24 h. Similarly, other drug-induced apoptosis also showed a similar tendency for cleaved-caspase expression. For example, cleaved-caspase 3 increased at 6–12 h and declined at 24 h in 0.2 μM staurosporine-treated human endothelial cornea cells [31]. After treatment of 0.2 μM staurosporine, cleaved-caspase 3 was also increased at 2–8 h and declined at 12–24 h for human cervical cancer HeLa cells. Cleaved-caspase 3 increased at 0.5–1 h and declined at 2–24 h in cervical cancer C-33A cells [32].

Mounting evidence demonstrated that ROS generation and mitochondrial membrane depolarization may lead to DNA damage [33,34,35,36,37,38] and apoptosis [38,39,40,41] in drug-treated cancer cells. Accordingly, TFB also showed a correlation between oxidative stress and DNA damage in its antiproliferative and apoptotic effects of two oral cancer cells (Ca9-22 and CAL 27) in our study.

## 4. Conclusions

TFB treatment induces apoptosis, ROS generation, mitochondrial depolarization, and DNA damage, which ultimately results in the antiproliferation of oral cancer Ca9-22 cells. This study also shows that TFB selectively kills the two oral cancer cell lines tested here and opts for its application in anti-oral cancer therapies.

## 5. Materials and Methods

### 5.1. Drug Information and Oral Cancer and Normal Cell Lines

The TFB (C_26_H_46_O_3_; MW: 406.3447; [3-(1-methoxyeicosyl)-5-methylene-5*H*-furan-2-one]) was purified from methanol extracts of *C. tenuifolium* stem as previously described [22]. It was dissolved in dimethyl sulfoxide (DMSO) for drug treatments. The oral cancer cell lines Ca9-22 [42] and CAL 27 [43] were incubated in a mixed medium with Dulbecco’s Modified Eagle Medium (DMEM) and F12 (Gibco, Grand Island, NY, USA) (3:2), 10% fetal bovine serum, antibiotics (penicillin and streptomycin), and others under a humidified atmosphere with 5% CO_2_ at 37 °C. The normal oral cells (human gingival fibroblasts, HGF-1) were incubated in a DMEM medium with a similar supplement with 1 mM pyruvate as described above.

### 5.2. Measurement of Cell Viability—Cellular ATP Content

Cellular ATP level was determined by using the ATP-lite Luminescence ATP Detection Assay System (PerkinElmer Life Sciences, Boston, MA, USA) according to the manufacturer’s instructions with a slight modification [44]. Briefly, Ca9-22 cells were plated at 4000 cells/well in 96-well plates. After seeding overnight, cells were treated with vehicle control (DMSO) or with TFB at indicated concentrations (5, 10, and 15 μM) for 24 h. After removing the medium solution, 100 μL of serum-free medium and 50 μL of a mammalian cell lysis solution was added per well of a microplate with orbital shaking at 100 rpm for 5 min. Then, 100 μL of the cell lysates/well was transferred to white 96-well plates and reacted with 50 μL of substrate solution (D-Luciferin and luciferase) under orbital shaking at 100 rpm for 5 min and then left to stand in darkness for a further 10 min. Finally, the luminescence was assayed using a microplate luminometer (CentroPRO LB 962, Berthold, ND, USA).

### 5.3. Measurement of Cell Cycle Progression

The cellular DNA was stained with PI as previously described [45]. Cells were plated at 3 × 10^5^ cells/2 mL cell culture medium on a 6-well plate. Briefly, cells were added with vehicle (DMSO only) or TFB. After collection for 70% ethanol fixation overnight, the centrifuged cell pellets were resuspended in 1 mL of PBS containing 50 μg/mL PI for 15 min at room temperature in darkness. Subsequently, these samples were examined using a FACSCalibur flow cytometer (Becton-Dickinson, Mansfield, MA, USA) (excitation: 488 nm and emission: 617 nm) and BD Accuri C6 software.

### 5.4. Measurement of Apoptosis by Annexin V Staining

Annexin V (Strong Biotech Corporation, Taipei, Taiwan)/PI (Sigma, St. Louis, MO, USA) double-staining for apoptosis analysis was performed as previously described [46]. Cells were plated at 3 × 10^5^ cells/2 mL cell culture medium on a 6-well plate with or without 0.1 mM Z-VAD-FMK pretreatments for 2 h (Selleckchem.com; Houston, TX, USA). Briefly, cells were added with vehicle or TFB. The cells were then resuspended in the binding buffer containing 5 μg/mL of annexin V-fluorescein isothiocyanate and 50 μg/mL of PI and examined with a BD Accuri C6 flow cytometer (Becton-Dickinson, Mansfield, MA, USA) (excitation: 488 nm and emission: 525 nm and 617 nm for FITC and PI, respectively) and BD Accuri C6 software.

### 5.5. Measurement of Apoptosis by Caspase Activity

Apoptosis was also measured by caspase activation [47]. The generic caspase activity assay kit (Fluorometric-Green; ab112130) (Abcam, Cambridge, UK) was used to detect the activity of caspase-1, -3, -4, -5, -6, -7, -8, and -9 as described [42]. Briefly, cells were seeded as 3 × 10^5^ cells per well in 6-well plates with a 2 mL medium for overnight. The cells were then treated with vehicle or TFB. Subsequently, cells were incubated at 37 °C, 5% CO_2_ for 2 h with 2 μL of 500X TF2-VAD-FMK. After PBS washing, cells were resuspended in 0.5 mL of an assay buffer for immediate flow cytometry measurement (BD Accuri™ C6; Becton-Dickinson).

The apoptosis signaling expressions were further measured via Western blotting. 30 μg protein lysates were resolved in 10% SDS-PAGE. After electrotransferring, the nonspecific bindings of PDVF membranes (Pall Corporation, Port Washington, NY, USA) were blocked with 5% non-fat milk in Tris-buffered saline with Tween-20 and incubated with primary antibodies (the cleaved caspase-8 (Asp391) (18C8) rabbit mAb and the rabbit mAb in the apoptosis antibody sampler kit (cleaved PARP (Asp214) (D64E10) XP^®^; cleaved caspase-3 (Asp175) (5A1E); cleaved caspase-9 (Asp330) (D2D4) from Cell Signalling Technology, Inc., Danvers, MA, USA, β-actin (#GTX629630, GeneTex Inc.)) under 1:10000 dilution as well as their matched secondary antibody. The WesternBright™ ECL HRP substrate (#K-12045-D50, Advansta, Menlo Park, CA, USA) was chosen for signal amplification.

### 5.6. Measurement of Intracellular ROS

2′,7′-Dichlorodihydrofluorescein diacetate (DCFH-DA) (Sigma Chemical Co., St. Louis, MO, USA) was used to detect intracellular ROS as previously described [33]. Cells were plated at 3 × 10^5^ cells/2 mL cell culture medium on a 6 cm dish. Briefly, cells were added with vehicle or TFB with or without 2 mM NAC pretreatment for 1 h (Sigma; St. Louis, MO, USA). After the collection and PBS washing, cells were treated with 0.1 μM DCFH-DA in serum-free medium for 30 min at 37 °C in darkness. Cells were resuspended in PBS after centrifugation and examined with a BD Accuri C6 flow cytometer (excitation: 488 nm and emission: 525 nm) and BD Accuri C6 software.

### 5.7. Measurement of MitoMP

A MitoProbe™ DiOC_2_(3) assay kit (Invitrogen, San Diego, CA, USA) was used to detect mitochondrial membrane potential (MitoMP) as described previously [34]. Cells were plated at 3 × 10^5^ cells/2 mL cell culture medium on a 6-well plate. Briefly, cells were added with vehicle or with TFB. The TFB-treated cells were washed in 1 mL of PBS/well, provided with 1 mL of medium/well, loaded with 10 μL of 10 μM DiOC_2_(3), and left to stand at 37 °C in 5% CO_2_ for 20–30 min. After harvesting and washing, cells were resuspended in PBS and examined immediately using a FACSCalibur flow cytometer (excitation: 488 nm and emission: 525 nm) and BD Accuri C6 software.

### 5.8. Measurement of DNA Damage by γH2AX Expression

DNA double strand breaks were detected by both flow cytometry [48] and Western blotting [35] as described previously. For flow cytometry, TFB-treated cells were fixed, washed, and incubated at 4 °C for 1 h in 2 μg/mL of p-Histone H2AX (Ser 139) (γH2AX) monoclonal antibody (sc-101696; Santa Cruz Biotechnology, Santa Cruz, CA, USA). After washing, cells were suspended for 1 h in a secondary antibody (Jackson Laboratory, Bar Harbor, ME, USA) for 30 min at room temperature. Finally, the cells were resuspended in 20 μg/mL of PI for flow cytometry analysis (BD Accuri™ C6; Becton-Dickinson).

For Western blotting of γH2AX expression, 30 μg protein lysates were resolved in 10% SDS-PAGE. Except when p-Histone H2AX (Santa Cruz Biotechnology) was chosen for the primary antibody, procedures were the same as those employing Western blotting for apoptosis proteins, mentioned above.

### 5.9. Statistical Analysis

All data are shown as mean ± SD. The significant differences between test and control were analyzed with a Student *t-*test.

## References

[1] Warnakulasuriya S. (2009). Global epidemiology of oral and oropharyngeal cancer. Oral Oncol..

[2] Ko Y.C., Huang Y.L., Lee C.H., Chen M.J., Lin L.M., Tsai C.C. (1995). Betel quid chewing, cigarette smoking and alcohol consumption related to oral cancer in Taiwan. J. Oral Pathol. Med..

[3] Lee C.H., Ko Y.C., Huang H.L., Chao Y.Y., Tsai C.C., Shieh T.Y., Lin L.M. (2003). The precancer risk of betel quid chewing, tobacco use and alcohol consumption in oral leukoplakia and oral submucous fibrosis in southern Taiwan. Br. J. Cancer.

[4] Chiang S.L., Lee C.P., Chang J.G., Lee C.H., Yeh K.T., Tsai Y.S., Chen M.K., Chen C.H., Ko Y.C. (2013). Combined effects of differentiation factor 15 and substance use of alcohol, betel quid and cigarette on risk of head and neck cancer. Head Neck Oncol..

[5] Myoung H., Hong S.P., Yun P.Y., Lee J.H., Kim M.J. (2003). Anti-cancer effect of genistein in oral squamous cell carcinoma with respect to angiogenesis and in vitro invasion. Cancer Sci..

[6] Chen B.H., Chang H.W., Huang H.M., Chong I.W., Chen J.S., Chen C.Y., Wang H.M. (2011). (−)-Anonaine induces DNA damage and inhibits growth and migration of human lung carcinoma h1299 cells. J. Agric. Food Chem..

[7] Wang H.M., Cheng K.C., Lin C.J., Hsu S.W., Fang W.C., Hsu T.F., Chiu C.C., Chang H.W., Hsu C.H., Lee A.Y. (2010). Obtusilactone A and (−)-sesamin induce apoptosis in human lung cancer cells by inhibiting mitochondrial Lon protease and activating DNA damage checkpoints. Cancer Sci..

[8] Hseu Y.C., Wu C.R., Chang H.W., Kumar K.J., Lin M.K., Chen C.S., Cho H.J., Huang C.Y., Lee H.Z., Hsieh W.T. (2011). Inhibitory effects of Physalis angulata on tumor metastasis and angiogenesis. J. Ethnopharmacol..

[9] Russo M., Spagnuolo C., Tedesco I., Russo G.L. (2010). Phytochemicals in cancer prevention and therapy: Truth or dare?. Toxins.

[10] Melchini A., Traka M.H. (2010). Biological profile of erucin: A new promising anticancer agent from cruciferous vegetables. Toxins.

[11] Yadav V.R., Prasad S., Sung B., Kannappan R., Aggarwal B.B. (2010). Targeting inflammatory pathways by triterpenoids for prevention and treatment of cancer. Toxins.

[12] Cabello C.M., Bair W.B., Lamore S.D., Ley S., Bause A.S., Azimian S., Wondrak G.T. (2009). The cinnamon-derived Michael acceptor cinnamic aldehyde impairs melanoma cell proliferation, invasiveness, and tumor growth. Free Radic. Biol. Med..

[13] Shu P., Wei X., Xue Y., Li W., Zhang J., Xiang M., Zhang M., Luo Z., Li Y., Yao G. (2013). Wilsonols A-L, megastigmane sesquiterpenoids from the leaves of *Cinnamomum wilsonii*. J. Nat. Prod..

[14] Chen C.Y., Chen C.H., Lo Y.C., Wu B.N., Wang H.M., Lo W.L., Yen C.M., Lin R.J. (2008). Anticancer activity of isoobtusilactone A from *Cinnamomum kotoense*: Involvement of apoptosis, cell-cycle dysregulation, mitochondria regulation, and reactive oxygen species. J. Nat. Prod..

[15] Chen C.Y., Liu T.Z., Chen C.H., Wu C.C., Cheng J.T., Yiin S.J., Shih M.K., Wu M.J., Chern C.L. (2007). Isoobtusilactone A-induced apoptosis in human hepatoma Hep G2 cells is mediated via increased NADPH oxidase-derived reactive oxygen species (ROS) production and the mitochondria-associated apoptotic mechanisms. Food Chem. Toxicol..

[16] Chen C.Y., Hsu Y.L., Chen Y.Y., Hung J.Y., Huang M.S., Kuo P.L. (2007). Isokotomolide A, a new butanolide extracted from the leaves of *Cinnamomum kotoense*, arrests cell cycle progression and induces apoptosis through the induction of p53/p21 and the initiation of mitochondrial system in human non-small cell lung cancer A549 cells. Eur. J. Pharmacol..

[17] Yang S.S., Hou W.C., Huang L.W., Lee T.H. (2006). A new gamma-lactone from the leaves of *Cinnamomum kotoense*. Nat. Prod. Res..

[18] Lin R.J., Lo W.L., Wang Y.D., Chen C.Y. (2008). A novel cytotoxic monoterpenoid from the leaves of *Cinnamomum subavenium*. Nat. Prod. Res..

[19] Yang S.Y., Wang H.M., Wu T.W., Chen Y.J., Shieh J.J., Lin J.H., Ho T.F., Luo R.J., Chen C.Y., Chang C.C. (2013). Subamolide B isolated from medicinal plant *Cinnamomum subavenium* induces cytotoxicity in human cutaneous squamous cell carcinoma cells through mitochondrial and CHOP-dependent cell death pathways. Evid Based Complement. Altern. Med..

[20] Liu C.H., Chen C.Y., Huang A.M., Li J.H. (2011). Subamolide A, a component isolated from *Cinnamomum subavenium*, induces apoptosis mediated by mitochondria-dependent, p53 and ERK1/2 pathways in human urothelial carcinoma cell line NTUB1. J. Ethnopharmacol..

[21] Chen T.H., Huang Y.H., Lin J.J., Liau B.C., Wang S.Y., Wu Y.C., Jong T.T. (2010). Cytotoxic lignan esters from *Cinnamomum osmophloeum*. Planta Med..

[22] Lin R.J., Cheng M.J., Huang J.C., Lo W.L., Yeh Y.T., Yen C.M., Lu C.M., Chen C.Y. (2009). Cytotoxic compounds from the stems of *Cinnamomum tenuifolium*. J. Nat. Prod..

[23] Chen H.L., Kuo S.Y., Li Y.P., Kang Y.F., Yeh Y.T., Huang J.C., Chen C.Y. (2012). A new benzodioxocinone from the leaves of *Cinnamomum tenuifolium*. Nat. Prod. Res..

[24] Tam K.F., Ng T.Y., Liu S.S., Tsang P.C., Kwong P.W., Ngan H.Y. (2005). Potential application of the ATP cell viability assay in the measurement of intrinsic radiosensitivity in cervical cancer. Gynecol. Oncol..

[25] Lu X., Errington J., Chen V.J., Curtin N.J., Boddy A.V., Newell D.R. (2000). Cellular ATP depletion by LY309887 as a predictor of growth inhibition in human tumor cell lines. Clin. Cancer Res..

[26] Dong H.P., Wu H.M., Chen S.J., Chen C.Y. (2013). The effect of butanolides from *Cinnamomum tenuifolium* on platelet aggregation. Molecules.

[27] Daker M., Lin V.Y., Akowuah G.A., Yam M.F., Ahmad M. (2013). Inhibitory effects of *Cinnamomum burmannii* Blume stem bark extract and trans-cinnamaldehyde on nasopharyngeal carcinoma cells; synergism with cisplatin. Exp. Ther. Med..

[28] Tait S.W., Green D.R. (2010). Mitochondria and cell death: Outer membrane permeabilization and beyond. Nat. Rev. Mol. Cell Biol..

[29] Chang Y.T., Huang C.Y., Li K.T., Li R.N., Liaw C.C., Wu S.H., Liu J.R., Sheu J.H., Chang H.W. (2016). Sinuleptolide inhibits proliferation of oral cancer Ca9–22 cells involving apoptosis, oxidative stress, and DNA damage. Arch. Oral Biol..

[30] Yen Y.H., Farooqi A.A., Li K.T., Butt G., Tang J.Y., Wu C.Y., Cheng Y.B., Hou M.F., Chang H.W. (2014). Methanolic extracts of *Solieria robusta* inhibits proliferation of oral cancer Ca9–22 cells via apoptosis and oxidative stress. Molecules.

[31] Thuret G., Chiquet C., Herrag S., Dumollard J.M., Boudard D., Bednarz J., Campos L., Gain P. (2003). Mechanisms of staurosporine induced apoptosis in a human corneal endothelial cell line. Br. J. Ophthalmol..

[32] Nicolier M., Decrion-Barthod A.Z., Launay S., Pretet J.L., Mougin C. (2009). Spatiotemporal activation of caspase-dependent and -independent pathways in staurosporine-induced apoptosis of p53wt and p53mt human cervical carcinoma cells. Biol. Cell.

[33] Yeh C.C., Yang J.I., Lee J.C., Tseng C.N., Chan Y.C., Hseu Y.C., Tang J.Y., Chuang L.Y., Huang H.W., Chang F.R. (2012). Anti-proliferative effect of methanolic extract of *Gracilaria tenuistipitata* on oral cancer cells involves apoptosis, DNA damage, and oxidative stress. BMC Complement. Altern. Med..

[34] Yen C.Y., Chiu C.C., Haung R.W., Yeh C.C., Huang K.J., Chang K.F., Hseu Y.C., Chang F.R., Chang H.W., Wu Y.C. (2012). Antiproliferative effects of goniothalamin on Ca9–22 oral cancer cells through apoptosis, DNA damage and ROS induction. Mutat. Res..

[35] Chiu C.C., Haung J.W., Chang F.R., Huang K.J., Huang H.M., Huang H.W., Chou C.K., Wu Y.C., Chang H.W. (2013). Golden berry-derived 4beta-hydroxywithanolide E for selectively killing oral cancer cells by generating ROS, DNA damage, and apoptotic pathways. PLoS ONE.

[36] Guo J., Zhao W., Hao W., Ren G., Lu J., Chen X. (2014). Cucurbitacin B induces DNA damage, G2/M phase arrest, and apoptosis mediated by reactive oxygen species (ROS) in leukemia K562 cells. Anticancer Agents Med. Chem..

[37] Ni C.H., Yu C.S., Lu H.F., Yang J.S., Huang H.Y., Chen P.Y., Wu S.H., Ip S.W., Chiang S.Y., Lin J.G. (2014). Chrysophanol-induced cell death (necrosis) in human lung cancer A549 cells is mediated through increasing reactive oxygen species and decreasing the level of mitochondrial membrane potential. Environ. Toxicol..

[38] Hseu Y.C., Lee M.S., Wu C.R., Cho H.J., Lin K.Y., Lai G.H., Wang S.Y., Kuo Y.H., Kumar K.J., Yang H.L. (2012). The chalcone flavokawain B induces G2/M cell-cycle arrest and apoptosis in human oral carcinoma HSC-3 cells through the intracellular ROS generation and downregulation of the Akt/p38 MAPK signaling pathway. J. Agric. Food Chem..

[39] Huang F.J., Hsuuw Y.D., Chan W.H. (2013). Characterization of apoptosis induced by emodin and related regulatory mechanisms in human neuroblastoma cells. Int. J. Mol. Sci..

[40] Shih H.C., El-Shazly M., Juan Y.S., Chang C.Y., Su J.H., Chen Y.C., Shih S.P., Chen H.M., Wu Y.C., Lu M.C. (2014). Cracking the cytotoxicity code: Apoptotic induction of 10-acetylirciformonin B is mediated through ROS generation and mitochondrial dysfunction. Mar. Drugs.

[41] Thangam R., Senthilkumar D., Suresh V., Sathuvan M., Sivasubramanian S., Pazhanichamy K., Gorlagunta P.K., Kannan S., Gunasekaran P., Rengasamy R. (2014). Induction of ROS-dependent mitochondria-mediated intrinsic apoptosis in MDA-MB-231 cells by glycoprotein from *Codium decorticatum*. J. Agric. Food Chem..

[42] Yeh C.C., Tseng C.N., Yang J.I., Huang H.W., Fang Y., Tang J.Y., Chang F.R., Chang H.W. (2012). Antiproliferation and induction of apoptosis in Ca9–22 oral cancer cells by ethanolic extract of *Gracilaria tenuistipitata*. Molecules.

[43] Jiang L., Ji N., Zhou Y., Li J., Liu X., Wang Z., Chen Q., Zeng X. (2009). CAL 27 is an oral adenosquamous carcinoma cell line. Oral Oncol..

[44] Wei J., Stebbins J.L., Kitada S., Dash R., Zhai D., Placzek W.J., Wu B., Rega M.F., Zhang Z., Barile E. (2011). An optically pure apogossypolone derivative as potent pan-active inhibitor of anti-apoptotic bcl-2 family proteins. Front. Oncol..

[45] Chiu C.C., Chang H.W., Chuang D.W., Chang F.R., Chang Y.C., Cheng Y.S., Tsai M.T., Chen W.Y., Lee S.S., Wang C.K. (2009). Fern plant-derived protoapigenone leads to DNA damage, apoptosis, and G(2)/m arrest in lung cancer cell line H1299. DNA Cell Biol..

[46] Chiu C.C., Liu P.L., Huang K.J., Wang H.M., Chang K.F., Chou C.K., Chang F.R., Chong I.W., Fang K., Chen J.S. (2011). Goniothalamin inhibits growth of human lung cancer cells through DNA damage, apoptosis, and reduced migration ability. J. Agric. Food Chem..

[47] Kaufmann S.H., Lee S.H., Meng X.W., Loegering D.A., Kottke T.J., Henzing A.J., Ruchaud S., Samejima K., Earnshaw W.C. (2008). Apoptosis-associated caspase activation assays. Methods.

[48] Yen C.Y., Hou M.F., Yang Z.W., Tang J.Y., Li K.T., Huang H.W., Huang Y.H., Lee S.Y., Fu T.F., Hsieh C.Y. (2015). Concentration effects of grape seed extracts in anti-oral cancer cells involving differential apoptosis, oxidative stress, and DNA damage. BMC Complement. Altern. Med..

